# A screening-level assessment of the pollinator-attractiveness of ornamental nursery stock using a honey bee foraging assay

**DOI:** 10.1038/s41598-020-57858-2

**Published:** 2020-01-21

**Authors:** Douglas B. Sponsler, Christina M. Grozinger, Rodney T. Richardson, Andrea Nurse, Dalton Brough, Harland M. Patch, Kimberly A. Stoner

**Affiliations:** 1 Department of Entomology, Pennsylvania State University, University Park, Pennsylvania, PA 16802 USA; 2 Department of Biology, York University, Toronto, ON M3J 1P3 Canada; 3 Climate Change Institute, University of Maine, 206 Sawyer Research Center, Orono, Maine 04469 USA; 4 The Connecticut Agricultural Experiment Station, 123 Huntington Street, New Haven, CT 06511 USA

**Keywords:** Evolutionary ecology, Behavioural ecology

## Abstract

In urban and suburban landscapes characterized by extensive designed greenspaces, the support of pollinator communities hinges significantly on floral resources provided by ornamental plants. The attractiveness of ornamental plants to pollinators, however, cannot be presumed, and some studies suggest that a majority of ornamental plant varieties receive little or no pollinator visitation. Here, we harness the sampling power of the western honey bee, a generalist pollinator whose diet breadth overlaps substantially with that of other pollinators, to survey the utilization of ornamental plants grown at three commercial nurseries in Connecticut, USA. Using a combination of DNA metabarcoding and microscopy, we identify, to genus-level, pollen samples from honey bee colonies placed within each nursery, and we compare our results with nursery plant inventories to identify the subset of cultivated genera that were visited during pollen foraging. Samples were collected weekly from May to September, encompassing the majority of the growing season. Our findings show that some plant genera known to be cultivated as ornamentals in our system, particularly ornamental trees and shrubs (e.g. *Hydrangea*, *Rosa*, *Spiraea*, *Syringa*, *Viburnum*), functioned as major pollen sources, but the majority of plants inventoried at our nurseries provided little or no pollen to honey bees. These results are in agreement with a growing body of literature highlighting the special importance of woody plants as resources for flower-visiting insects. We encourage further exploration of the genera highlighted in our data as potential components of pollinator-friendly ornamental greenspace.

## Introduction

In an incisive critique, Quigley^1^ characterized ornamental greenspaces as “Potemkin gardens”: mere ecological facades, achieving a superficial green aesthetic while remaining wholly dependent upon ongoing human input and participating negligibly in the ecological communities upon which they are superimposed. While there may be many ways to assess the ecological function of ornamental flora, their capacity to function in plant-animal mutualisms is perhaps the most far-reaching in its significance^2^. A special case of this function is the provisioning of floral resources to pollinators: organisms, principally insects, that subsist on pollen and/or nectar and that facilitate plant reproduction by the transport of pollen during their serial visitation of flowers. Many pollinator populations around the world are in decline, and several species are threatened or endangered^3^. The availability of floral resources is an important constraint on pollinator populations^4^, and observed declines are likely driven to a large extent by the loss of foraging habitat^5^.

In apparent conflict with Quigley’s^1^ characterization of ornamental greenspaces is the observation that urban landscapes, which tend to be enriched in ornamental greenspaces, can support diverse pollinator communities^6^, and within cities it may be precisely the ornamental greenspaces where the highest density of pollinators can be found^7^. Nevertheless, while ornamental plants are characteristically “showy” in their floral displays, their attractiveness to pollinators cannot be presumed. Ornamental cultivars are bred, often by means of hybridization and asexual propagation, to generate phenotypes that appeal to human aesthetics and facilitate horticultural production^8^, resulting in a recent evolutionary history devoid of selection pressure toward pollinator attraction and reward. In this artificial evolutionary process, traits involved in attracting pollinators may be lost, gained, or altered, with corresponding effects on the presence, attractiveness, and accessibility of floral rewards for pollinators^9,10^. Previous studies have shown that the attractiveness of ornamental plants to pollinators is highly variable and often heavily skewed such that a minority of varieties are disproportionately attractive while the majority attract few or no visitors^11–14^, though attractive ornamentals in a given locality can receive extensive pollinator visitation^15^. Thus, with respect to trophic interaction with pollinators, it might be said that evaluating the significance of ornamentals involves identifying the functional minority within the merely decorative majority.

To address this question, we used the pollen and nectar generalist western honey bee (*Apis mellifera* L.). While pollinator species can differ markedly in floral diet, the honey bee is an extreme generalist whose diet breadth overlaps substantially with that of the larger pollinator community^16^, and its large foraging range and amenability to standardized sampling techniques make it uniquely suited for landscape-scale assays of floral resource use. Here, we report a study of honey bee foraging at three sites within large ornamental plant nurseries. Using DNA metabarcoding cross-validated with microscopy, we identify, to genus level, the floral sources of pollen samples collected throughout the foraging season. By comparing these results to inventories of genera cultivated by the nurseries at our study sites–with the caveat that ornamental species and cultivars cannot, at genus-level resolution, be distinguished definitively from wild congeners–we identify plausibly ornamental genera (i.e. genera known to have been cultivated at our nursery sites) that attracted substantial honey bee foraging. We interpret these findings as a screening-level survey of ornamental flora that merit consideration in the design of greenspaces aiming to reconcile human aesthetics and pollinator provisioning.

## Results

### Metabarcoding results

Across our 47 samples, we obtained 518,475 mate-paired reads successfully aligned with ITS2 reference sequences. After trimming to 95% identity and 300 bp alignment length, 432,954 alignments (84%) were retained.

**Figure 1:**
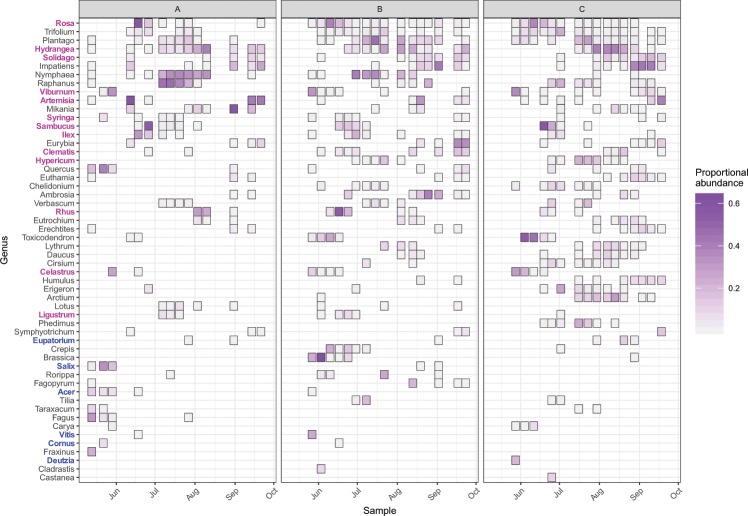
Major genera detected by metabarcoding. Any genera present at 5% of higher proportional abundance within at least one of the samples are considered “major genera” and are presented in this figure. Genera known to be cultivated in one or more of the nurseries used in this study are shown in bold typeface: blue for genera found only in the Nursery B inventory, pink for genera found in the inventories of both Nursery B and C. Note that we did not have information on the genera present in Nursery A’s inventories. Genera are ordered top to bottom by decreasing frequency of detection. Proportional abundance is defined as the number of reads aligned to a given genus in a given sample divided by the total number of reads aligned in a given sample.

A total of 141 genera were detected. Of these, 54 comprised at least 5% of at least one sample and were thus considered “major” genera. Nineteen major genera, including 5 of the top 10 most frequently detected genera, were known to have been cultivated in one or more of the nursery study sites (Fig. 1). For full, tabulated results, see Supplemental Material.

### Microscopy results

**Figure 2:**
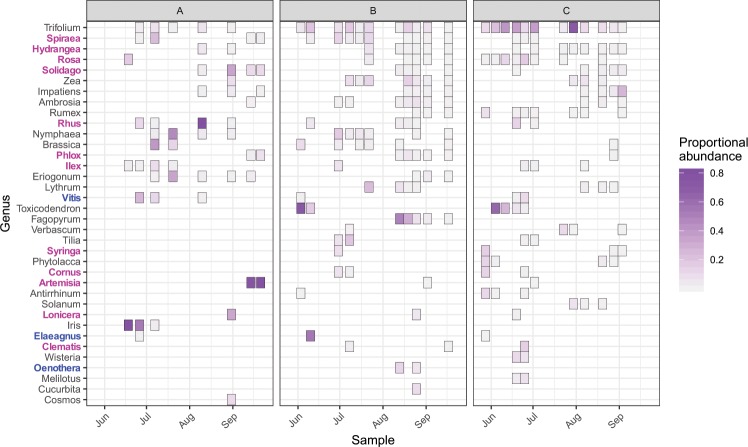
Major genera detected by microscopy. Genera known to be cultivated in one or more of the nurseries used in this study are shown in bold typeface: blue for genera found only in the Nursery B inventory, pink for genera found in the inventories of both Nursery B and C. Genera are ordered top to bottom by decreasing frequency of detection. Proportional abundance is defined as the pollen grain volume of a given genus in a given sample divided by the cumulative pollen grain volume a given sample.

Thirty-one of our 47 samples were examined using microscopy. A total of 101 genera were detected by microscopy. Of these, 36 met the criterion for major genera. Fifteen major genera, including 5 of the top 10 most frequently detected genera, were known to have been cultivated in one or more of the nursery study sites (Fig. 2). For full, tabulated results, see Supplemental Material.

### Comparison of metabarcoding and microscopy

**Figure 3:**
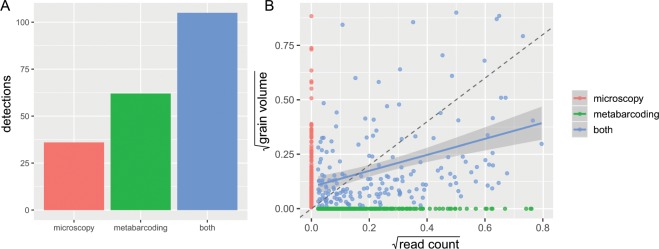
Comparison of metabarcoding and microscopy in terms of genus-by-sample detections (**A**) and genus-by-sample abundance (**B**). Analysis of genus detections (**A**) is limited to major genera (≥5% abundance in at least one method). The y-axis depicts the number of genus detections across all samples for which both microscopy and metabarcoding data were available (many genera were detected more than once). Analysis of abundance (**B**) is not limited to major genera. The linear regression (blue line with standard error) was fit only to genera detected by both methods. A dashed y = x line is shown for comparison. Both axes have been square-root-transformed.

Across the 31 samples for which complementary metabarcoding and microscopy data were available, 62 genus detections (30%) were made by metabarcoding but not by microscopy, 36 genus detections (18%) were made by microscopy but not by metabarcoding, and 105 genus detections were made by by both (52%) (Fig. 3). For genus detections shared by both methods (“shared detections”), there was a positive but very noisy linear relationship (F = 30.89, adjusted R^2^ = 0.14, P = 9.8 × 10^−8^) between read count (metabarcoding) and grain volume (microscopy) (Fig. 3).

Six of the 36 genera detected by microscopy but not by metabarcoding (*Cytisus*, *Equisetum*, *Eriogonum*, *Liatris*, *Nyssa*, and *Securigera*) were absent from the ITS2 reference database and thus undetectable by metabarcoding. Of these, only *Eriogonum* qualified as a major genus in the microscopy data set.

### Comparison of genera detected with genera in nursery inventories

**Figure 4:**
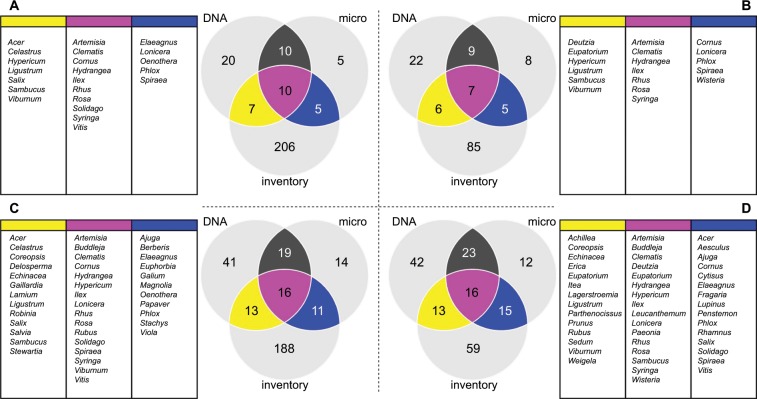
Comparison of genera detected by metabarcoding and microscopy with those present in nursery inventories: Nursery B major genera (**A**), Nursery C major genera (**B**), Nursery B all genera (**C**), Nursery C all genera. (**D**) Venn diagrams depict the numerical overlap in genera. Beside each Venn diagram, genera observed by either method and present in the nursery inventory are listed in boxes with headers colored to match their corresponding Venn diagram compartments.

Nursery B’s inventory contained a total of 228 genera. Of these, 40 were detected in our pollen samples (Fig. 4C), and 22 qualified as major genera (Fig. 4A). Nursery C’s inventory contained a total of 103 genera. Of these, 44 were detected in our pollen samples (Fig. 4D), and 18 qualified as major genera (Fig. 4B).

## Discussion

### Taxonomic composition of pollen samples

An important caveat for this discussion is that genus-level identification cannot determine conclusively whether, in our study system, a given taxon represents a cultivated ornamental plant. Indeed, all of the putative ornamentals that occur as “major genera” in our samples have native or naturalized representatives in our study region^17^, and the kilometer-scale foraging range of honey bees^18^ extends well beyond the bounds of our nursery sites. Thus, the provenance of each genus in our dataset should be interpreted cautiously. The occurrence of *Acer*, *Artemesia*, *Celastrus*, *Eupatorium*, *Rhus*, *Salix*, *Solidago*, and *Vitis* in our samples should be attributed primarily to foraging on wild plants, since wild representatives of these genera occur abundantly in our study region and most are known to attract honey bee foraging^19^. For the remainder of the putatively ornamental genera in our dataset, their occurrence can plausibly be attributed to some combination of foraging on nursery stock, cultivated or naturalized ornamental plants in the surrounding landscape, and wild congeners. In some cases, the known phenology of wild congeners can help parse the data. For example, the genus *Rosa* was the most frequently detected genus in our metabarcoding data set, but it occurs commonly both as ornamental cultivars and wild congeners. In our study region, wild *Rosa* is represented primarily by invasive *Rosa multiflora*, which blooms in June. Thus, *Rosa* detected in June, when peak *Rosa* abundance was observed in our samples, probably represents mostly wild *R*. *multiflora*, while the *Rosa* detected July-October probably represents mostly ornamental cultivars.

With respect to our central question of whether and to what extent ornamental plants function as resources for honey bees, the most salient pattern in our data is that only a minority of the genera known to be cultivated ornamentally in our study system contributed significantly to honey bee pollen foraging, but a few of these genera emerged as exceptionally important. This attractive minority consisted almost exclusively of trees, shrubs, or woody vines (e.g. *Hydrangea*, *Ilex*, *Rosa*, *Sambucus*, *Spiraea*, *Syringa*, *Viburnum*). Consistent with past studies^11–14^, ornamental forbs were, with few exceptions (e.g. *Phlox*), of marginal importance. These findings corroborate a growing body of literature establishing the special importance of trees and shrubs, including ornamental varieties^20^, in supporting pollinators^21–23^. That ornamental trees and shrubs can be effective alongside wild species invites the adoption of Donkersley’s^24^ “trees for bees” maxim in designed greenspaces where woody ornamentals might deliver ecologically functional aesthetics. A caveat to this interpretation of our data is that woody plants were represented disproportionately in the inventories of the nurseries in our study system (Supplemental Information), comprising 84% and 75% of individual plants at Nurseries B and C respectively. So, we cannot separate intrinsic attractiveness from mere resource volume. While this confounding relationship between attractiveness and volume is especially pronounced in our study system, it is inherent to both semi-natural and designed floral landscapes that feature trees and shrubs because the three-dimensional, spheroid growth form of these plants allows them to bear, all else held equal, more inflorescences per area of terrestrial footprint than low-growing herbaceous plants that comprise an essentially two-dimensional surface^24^.

It is worth noting the conspicuous absence of the family Ericaceae in both metabarcoding and microscopy datasets despite being represented by abundant plantings of *Rhododendron* at all our nursery sites. The pollen of *Rhododendron* is carried on viscin threads that bind together individual pollen grains and make it difficult for bees to release pollen from the anthers and manipulate it into pollen loads^25^. Some *Rhododendron* species can be highly attractive to honey bees, but the recovery of *Rhododendron* pollen from pollen traps would be unlikely even in circumstances when bees are visiting it for nectar.

Both metabarcoding and microscopy attest to the importance of *Nymphaea* (water lily) as major pollen source from early July to early August. Not mentioned in Ayers and Harman’s^19^ survey of honey and pollen plants, *Nymphaea* has perhaps been overlooked as a significant floral resource for honey bees, and probably other pollinators, including *Lasioglossum* sweat bees^26^. While this genus was not cultivated in any of our nurseries and grows natively in our study region, it is often used as an ornamental in landscapes featuring ponds or water gardens. The prevalence of *Nymphaeae* in our samples suggests the intriguing possibility of designed water features as pollinator habitat. “Pollinator ponds” would have the potential to provide floral resources, such as *Nymphaea*, during the sparse weeks of high summer^18^ while also providing water needed by honey bees (and possibly other species) for nest thermoregulation and brood rearing^27^.

Our data also showcase the importance of a diversity of wild plant taxa. Spring foraging was dominated by trees such as *Acer*, *Fagus*, *Quercus*, and *Salix*. In summer, herbaceous “weeds” like clovers and sweetclovers (*Melilotus*, *Trifolium*), mustards (*Brassica*, *Raphanus*), and plantain (*Plantago*) rise to prominence alongside woody plants like *Ilex*, *Rhus*, and *Toxicodendron*. In late summer and fall, a diversity of asters (*Ambrosia*, *Artemisia*, *Erechtites*, *Erigeron*, *Eupatorium*, *Eurybia*, *Euthamia*, *Eutrochium*, *Symphyotrichum*, *Solidago*) close out the foraging season.

It must be noted that our data pertain only to pollen foraging. Some plants can be major resources for nectar foraging while contributing minimally to pollen foraging^19,28^. Conversely, some of the plants we observed as major pollen sources, particularly the wind-pollinated genera (e.g. *Ambrosia*, *Artemisia*, *Carya*, *Castanea*, *Fagus*, *Fraxinus*, *Quercus*, *Rumex*), likely contributed little or no nectar^19^. Our data also pertain directly only to honey bee foraging. While honey bee foraging overlaps substantially with other bees^16^, foraging preferences can vary markedly even between the honey bee and other generalist bee species^29^, and the role of the honey bee as a proxy for non-bee insect pollinators would be still more tentative. Thus, our results should be interpreted conservatively with respect to the overall pollinator community, and future work focusing on non-bee pollinators would be especially warranted. Finally, without knowing the relative abundance, inflorescence density, or per-inflorescence reward of the plants detected in our study, we cannot abstract taxonomic “preference” from a general bias toward plants offering a favorable density of reward.

There is growing evidence that the pollinator attractiveness of ornamental plants can vary as much between cultivars of the same species as between representatives of different species (E. Erickson, personal communication). With conventional pollen metabarcoding, even species-level resolution is elusive, and it is doubtful that cultivar-level resolution would be consistently feasible with currently available markers. The nascent methods of long-read DNA metabarcoding^30^ and whole plastome genotyping^31^, however, may soon enable species- and cultivar-level identification of pollen, which will make possible the exploration of questions beyond the reach of current techniques.

### Comparison of metabarcoding and microscopy

The 52% agreement between metabarcoding and microscopy in genus-by-sample detections for major genera is comparable to past studies (e.g.^32,33^. Some of the unresolved disagreement between the two methods is likely due to sampling effects, particularly for microscopy, where slides were prepared from small (0.47–0.85 g) bulk samples of corbicular pollen compared with the 4 g bulk samples of corbicular pollen used for DNA extraction. In other cases, though, shortcomings seem to have been inherent to the respective methods. Metabarcoding, for example, seems to have greatly underestimated the abundance of *Spiraea* and completely missed *Elaeagnus* and *Iris* despite these taxa being represented in the reference database. Whether these artifacts are due to negative PCR bias or taxonomic misassignment is uncertain. On the other hand, metabarcoding seems to have done a better job resolving genera within the diverse Asteraceae family (i.e. *Ambrosia*, *Arctium*, *Artemisia*, *Cirsium*, *Erechtites*, *Erigeron*, *Eurybia*, *Euthamia*, *Eutrochium*, *Mikania*, *Solidago*, *Symphyotrichum*, *Taraxacum*, *Verbascum*) whose cryptic pollen morphology presents a greater challenge to microscopy.

Considering only the subset of shared genus detections, the quantitative relationship between the two methods was statistically significant, but too noisy to be considered predictive for any particular observation, consistent with past studies that have found ITS2 to be quantitatively problematic as a pollen metabarcoding marker^22,34^. Since ITS2 performs more reliably for inferring presence/absence than abundance, the frequency with which a given taxon occurs across samples is a more robust measure of dietary significance.

## Conclusion

Using the honey bee as a model generalist pollinator, we found evidence that some ornamental flora, particularly trees and shrubs, can function alongside wild plants as major contributors to pollen foraging. The prominent role of *Nymphaea* in our study suggests that floricultural water features have been under-appreciated for their potential to support pollinators. Future studies of floricultural pollinator habitat should focus on wild pollinators and explore the role of ornamental plants in structuring plant-pollinator networks^15^. Refinement of our preliminary assay will also require more complete knowledge of foraging habitat to enable the definitive delineation of ornamental taxa and the parsing of intrinsic attractiveness and resource volume.

Behind the question of ornamental plants as pollinator resources lies the larger issue of reconciling human land use with conservation and ecosystem function^35^. The provisioning of pollinators is one of many angles from which this issue may be approached, and our findings should not be interpreted in isolation. Tallamy’s^36^ work, for example, emphasizes the trophic role of plants in supporting higher trophic levels through the provisioning of edible foliage, a role for which many exotic ornamentals may be poorly suited irrespective of their floral attractiveness. Finally, it is worth noting that while ornamental plants comprise a minor component of most landscapes, they play a disproportionate role in mediating the relationship between flower-visiting insects and people. In this capacity, ornamental greenspaces that hum with bees might remedy the “extinction of experience” that threatens to dull the human impetus to conservation^37^.

## Methods

### Study sites and sampling regime

The current study used samples that were previously analyzed for pesticide residues and reported in^38^. The study sites and pollen sampling scheme are described in detail in^38^, and briefly described here.

Our study sites were three wholesale plant nurseries in Connecticut, USA, separated from each other by a minimum of 42 km. Nursery A (48 ha) is located in south-central Connecticut, 2.4 km from Long Island Sound, and surrounded by forest interspersed with suburban development. Nursery B (183 ha) is located in north-central Connecticut and is surrounded by agricultural fields, suburban development, and forest. Nursery C (168 ha) is located in a rural area of eastern Connecticut surrounded primarily by a mix of agricultural fields and forest. All three nurseries rely heavily on rhododendrons and azaleas for their business, but otherwise varied in their product mix. We obtained inventory lists from Nurseries B and C to establish which genera were cultivated at our study sites (Supplementary Information). We were unable to obtain an inventory from Nursery A, so this site was omitted when comparing our samples with nursery inventories (Fig. 4).

Nine honey bee colonies (*Apis mellifera carnica* L.) were started from 3 lb packages, allowed to establish at the CT Agricultural Experiment Station Lockwood Farm (Hamden, CT), and then moved to each of the three commercial ornamental plant nurseries on May 8, 2015, resulting in three study colonies per nursery. Colonies were inspected weekly at the time of pollen collection to verify the presence of a laying queen, to prevent swarming, and to ensure proper functioning of the pollen traps. No treatments for mites or disease were applied during sampling season. Replacement queens (in cases of queen failure) and additional hive equipment (to accommodate colony growth) were added as needed.

All hives were equipped with Sundance I bottom-mounted pollen traps (Ross Rounds, Inc., Canandaigua, NY), which can be switched “on” or “off” to trap pollen or allow bees to pass unhindered, respectively. Pollen samples were collected on a weekly basis from May 8 to September 23, 2015. To reduce nutritional stress, the pollen trap on each hive was turned “on” (trapping pollen) for two weeks at a time, and then turned “off” for the third week to allow the bees to recover. This pattern of trapping and resting was staggered across the three hives at each site such that on each sampling date pollen could be harvested from two of the three hives. During sampling, pollen was collected separately from each hive using 50-ml centrifuge tubes, and excess pollen was collected into plastic bags to empty the traps for the next sampling period. Upon returning to the lab, pollen was frozen at −20 °C until further processing. Pooling samples across hives within each site-date yielded a total of 47 samples.

### Sample preparation

To subsample small amounts of pollen from corbicular pollen samples, we broke up the corbicular pollen pellets into a uniform mixture of individual pollen grains, a process that we term “granularization”. Working in 50 mL centrifuge tubes, 4 g of each sample of corbicular pollen (pooled by site-date) were suspended in 40 mL of 70% EtOH. The sample was then vortexed on high for 30 seconds. After vortexing, the pollen was left to settle before vortexing again for another 30 seconds and repeating as necessary until the pollen pellets were fully dispersed. The solution was then centrifuged (Eppendorf 5810 R, Hamburg, Germany) at 3000 G for 2 minutes and the supernatant discarded. The pollen sediment was then resuspended in 10 mL of 100% EtOH, and the vortexing and centrifugation steps were repeated a second time. Finally, the sample was left uncapped for 24 hours in order to allow the EtOH to evaporate and yield the final granularized pollen sample.

### DNA extraction

From each granularized sample, we transferred a 10 mg aliquot to a 2 mL beadmill tube (BioSpec, Bartlesville, OK) and added 1 mL of lysis buffer (buffer AP1; QIAGEN, Venlo, Netherlands) and ~0.5 mL of chrome steel beads (1.3 mm diameter; BioSpec, Bartlesville, OK). We then processed the samples on an Omni Bead Ruptor 24 Elite beadmill (Omni International, Kennesaw, GA) using a speed of 6 m/s and repeating for four 1-min runs, cooling samples on ice between runs. The resulting lysate was then purified using a Qiagen DNeasy Minikit according to the manufacturer’s protocol, and DNA yield was verified on a NanoDrop spectrophotometer (Thermo Fisher Scientific, Waltham, MA). Importantly, our choices of bead size, bead material, and beadmill run settings were based on preliminary trials in which we observed that beadmill homogenization with other combinations of parameters did not necessary lyse all grains and often lysed grains in a taxon-biased manner.

### Library construction and sequencing

For a barcoding marker, we chose the nuclear ITS2 region, which is a commonly used as a pollen barcoding marker (e.g.^39^). To amplify this region, we used the angiosperm-specific primers ITS2-An3 and ITS2-An4 described by Cheng *et al*.^40^. The plastid markers *rbcL* and *trnL* were also explored but were found to provide insufficient genus-level resolution in our study system, leading us to rely on ITS2 as a single-marker barcode.

Following Richardson *et al*.^34^, amplicon libraries were created using a nested PCR protocol designed to minimize taxonomic bias introduced by template-specific indexed primer interactions^41^. In PCR 1, the ITS2 target region was amplified using the primers described above. In PCR 2, Illumina read-priming oligos were added. In PCR 3, sample-specific dual-indices^39,42^ and Illumina hybridization oligos were added. We then cleaned and normalized the product of PCR 3 using the SequalPrep kit (Thermo Fisher Scientific). Finished libraries were sequenced at the Penn State Genomics Core Facility on the Illumina MiSeq platform (Illumina, Inc) using the 2 × 250 cycle Nano flow cell. Data are archived on NCBI, accession number PRJNA548313.

### Bioinformatics

Reference sequences were downloaded from GenBank using the eDirect API using the following search terms:

esearch -db nucleotide -query "Viridiplantae [ORGN] AND

(its2 OR internal transcribed spacer [ALL])" |

efilter -query 100:2000[SLEN]

Taxonomic lineages for each downloaded accession were retrieved using the R package taxonomizr^43^. Reference sequence sets were then trimmed to the precise barcode region of interest for each marker using MetaCurator^44^. The MetaCurator pipeline uses hidden Markov models, produced by HMMER3^45^, to identify and excise the amplicon region of interest from all available references, including long genomic contigs and whole plastid genomes. Additionally, using a VSEARCH-based method^46^, this procedure removes duplicate reference sequences using a taxonomically aware method wherein a duplicate is only removed from the data if it belongs to the same taxonomic lineage as the alternate duplicate. Finally, the curated database was geographically filtered at the genus level using USDA PLANTS Database^17^, records for the states of Connecticut, Massachusetts, New Jersey, New York, Pennsylvania, and Rhode Island, plus the genera found in our nursery inventories (some of which lacked USDA records for our study region). The final ITS2 database contained 43,844 sequences representing 1,183 of of the 1,994 genera in the USDA plant records (59%), plus 211 of the 247 genera from our nursery inventories (85%).

MiSeq paired-end reads were first mate-paired using PEAR^47^. Then, using the–usearch_global function of the VSEARCH program^46^, mate-paired reads were queried against the reference libraries described above, and we selected the top hit (i.e. best percent identity) for each query sequence after constraining hits to a minimum of 75% identity and 80% query coverage. The output of the–usearch_global alignment was then subjected to a more stringent 95% identity threshold and 300 bp alignment length threshold. All query sequences with alignments below identity threshold were considered unclassified.

While some studies have reported species-level taxonomic assignments using pollen metabarcoding, this approach is only appropriate for study systems with a regionally exhaustive reference library for which the species level resolution has been verified for the marker used. The construction of such a reference library was beyond the scope of this study, so we opted for the more conservative approach of assigning taxonomy only to the genus level. The inferred taxonomic composition of each sample was summarized by summing the total read counts by genus and excluding genera comprising less than 0.05% of the total reads as likely false positives.

Reference library curation and VSEARCH alignments were performed on high-performance computing clusters at the Penn State Institute for CyberSciences and the Ohio Supercomputer Center. Post-alignment data processing was performed in R^48^, and annotated scripts are available at https://github.com/sponslerdb/vsearchr.

### Pollen microscopy

Thirty-one of our 47 samples were cross-validated by microscopy to provide a second line of evidence for inferring the role of ornamental plants in the honey bee pollen diet. Pollen microscopy was performed at the Paleoecology Laboratory, Climate Change Institute, University of Maine, Orono, Maine, as described in^38^. Bulk corbicular pollen samples (0.47–0.85 g) were first granularized in 10% hydrochloric acid, washed in deionized water to remove excess organics, and then rinsed in glacial acetic acid to remove all water. Samples were then acetolyzed following^49^, mounted, and viewed at 400x magnification on a Nikon Labophot-2 microscope (Nikon Corporation, Tokyo). Pollen grains were identified to the lowest possible taxonomic level using reference specimens collected from the study nurseries, previously collected reference material at the Climate Change Institute, published keys^49–51^, and images from the PalDat database (https://www.paldat.org/). The proportional abundance of each pollen type was estimated using the volumetric methods of^52^.

### Data analysis

To simplify data visualization and focus interpretation on the most significant components of the honey bee pollen diet, we created subsets of our metabarcoding and microscopy data including only genera with a maximum proportional abundance within a site-date sample of at least 5%, hereafter termed “major genera”. These subsets of major genera were used to evaluate our central question of which ornamental plants were attractive to honey bees, as well as to evaluate the qualitative (presence/absence) agreement between metabarcoding and microscopy. For evaluating the quantitative consistency of metabarcoding and microscopy, we used the full datasets.

For the subset of genera within each sample detected by both metabarcoding and microscopy, we used linear regression to evaluate the quantitative consistency of the two methods using square-root transformed to improve homogeneity of variance. All processing and analysis was done in R^48^.

## Supplementary information

inventory_nurseryB.

inventory_nurseryC.

metabarcoding_data.

microscopy_data.
